# Muscle histopathology in *nebulin*-related nemaline myopathy: ultrastrastructural findings correlated to disease severity and genotype

**DOI:** 10.1186/2051-5960-2-44

**Published:** 2014-04-12

**Authors:** Edoardo Malfatti, Vilma-Lotta Lehtokari, Johann Böhm, Josine M De Winter, Ursula Schäffer, Brigitte Estournet, Susana Quijano-Roy, Soledad Monges, Fabiana Lubieniecki, Remi Bellance, Mai Thao Viou, Angéline Madelaine, Bin Wu, Ana Lía Taratuto, Bruno Eymard, Katarina Pelin, Michel Fardeau, Coen AC Ottenheijm, Carina Wallgren-Pettersson, Jocelyn Laporte, Norma B Romero

**Affiliations:** 1 Unité de Morphologie Neuromusculaire, Institut de Myologie, Groupe Hospitalier Universitaire La Pitié-Salpêtrière, Paris, France; 2 Department of Neurological, Neurosurgical, and Behavioral Sciences, University of Siena, Siena, Italy; 3 Inserm, U974, Paris F-75013, France; 4 Université Pierre et Marie Curie- Paris 6, UM 76, Inserm, U974, CNRS, UMR 7215, Institut de Myologie, Paris F-75013, France; 5 Centre de référence de Pathologie Neuromusculaire Paris-Est, Institut de Myologie, GHU La Pitié-Salpêtrière, Assistance Publique-Hôpitaux de Paris, Paris, France; 6 Department of Medical Genetics, Haartman Institute, University of Helsinki, and the Folkhälsan Institute of Genetics, Biomedicum Helsinki, Helsinki, Finland; 7 Department of Translational Medicine, IGBMC, INSERM U964, UMR7104, Strasbourg University, Illkirch, France; 8 Department of Physiology, VU University medical center, Amsterdam, the Netherlands; 9 AP-HP, Service de Pédiatrie, Hôpital Raymond Poincaré, Garches; Hôpitaux, Universitaires Paris-Ile-de-France Ouest, Pôle pédiatrique; Centre de Référence, Maladies Neuromusculaires Garches-Necker-Mondor-Hendaye (GNMH), Université Versailles Saint-Quentin en Yvelines (UVSQ), Garches, France; 10 Hospital Nacional de Pediatría J.P. Garrahan, and Instituto de Investigaciones Neurologicas, FLENI, Buenos Aires, Argentina; 11 CHU de Fort de France, Martinique, France; 12 BGI-Shenzhen, Shenzhen 518083, China; 13 Department of Biosciences, Division of Genetics, University of Helsinki, Helsinki, Finland

**Keywords:** Congenital myopathies, Nemaline myopathy, Nebulin, Muscle contractility

## Abstract

Nemaline myopathy (NM) is a rare congenital myopathy characterised by hypotonia, muscle weakness, and often skeletal muscle deformities with the presence of nemaline bodies (rods) in the muscle biopsy. The nebulin (*NEB*) gene is the most commonly mutated and is thought to account for approximately 50% of genetically diagnosed cases of NM. We undertook a detailed muscle morphological analysis of 14 *NEB*-mutated NM patients with different clinical forms to define muscle pathological patterns and correlate them with clinical course and genotype. Three groups were identified according to clinical severity. Group 1 (n = 5) comprises severe/lethal NM and biopsy in the first days of life. Group 2 (n = 4) includes intermediate NM and biopsy in infancy. Group 3 (n = 5) comprises typical/mild NM and biopsy in childhood or early adult life. Biopsies underwent histoenzymological, immunohistochemical and ultrastructural analysis. Fibre type distribution patterns, rod characteristics, distribution and localization were investigated. Contractile performance was studied in muscle fibre preparations isolated from seven muscle biopsies from each of the three groups. G1 showed significant myofibrillar dissociation and smallness with scattered globular rods in one third of fibres; there was no type 1 predominance. G2 presented milder sarcomeric dissociation, dispersed or clustered nemaline bodies, and type 1 predominance/uniformity. In contrast, G3 had well-delimited clusters of subsarcolemmal elongated rods and type 1 uniformity without sarcomeric alterations. In accordance with the clinical and morphological data, functional studies revealed markedly low forces in muscle bundles from G1 and a better contractile performance in muscle bundles from biopsies of patients from G2, and G3.

In conclusion *NEB*-mutated NM patients present a wide spectrum of morphological features. It is difficult to establish firm genotype phenotype correlation. Interestingly, there was a correlation between clinical severity on the one hand and the degree of sarcomeric dissociation and contractility efficiency on the other. By contrast the percentage of fibres occupied by rods, as well as the quantity and the sub sarcolemmal position of rods, appears to inversely correlate with severity. Based on our observations, we propose myofibrillar dissociation and changes in contractility as an important cause of muscle weakness in *NEB*-mutated NM patients.

## Introduction

Nemaline myopathy (NM) is a congenital muscle disorder associated with hypotonia, muscle weakness, and often skeletal muscle deformities with the presence of numerous nemaline bodies (rods) in muscle biopsy [1]. Clinically the disorder has a marked clinical variability, ranging from neonatal lethal to mild non-progressive forms with onset in childhood and adulthood. NM has been classified into six clinical categories according to the severity of the disease, the age of onset and the pattern of muscle weakness [2]. To date at least nine genes have been implicated in NM (*ACTA1*, MIM#161800; *NEB*, MIM#256030; *TPM2*, MIM#609285; *TPM3*, MIM#609284; *TNNT1*, MIM#605355; *KBTBD13*, MIM#609273; *CFL2*, MIM#610687; *KLHL40* MIM#615340; and *KLHL41*) encoding proteins of the thin filament of skeletal muscle sarcomere or the Kelch domain associated proteins [3-11]. *ACTA1, TPM2* and *TPM3* NM is inherited both as autosomal dominant or recessive trait, with de novo dominant mutations being common in all three genes. *KBTBD13* NM is an autosomal dominant disorder. The other five genes present autosomal recessive mode of inheritance.

Nebulin is a sarcomeric structural protein crucial for the proper assembly and function of thin filaments [12]. One molecule spans nearly the entire length of the thin filament, making nebulin one of the largest polypeptides in nature. The *nebulin* (*NEB*) gene is the most commonly mutated and is thought to account for approximately 50% of genetically diagnosed cases of NM [12]. It is composed by 183 exons of which at least 17 [13] have been shown to be alternatively spliced, giving rise to several different nebulin isoforms in skeletal muscle [14]. Molecular diagnosis has mostly been based on dHPLC and confirmed by exon Sanger sequencing which are time-consuming, laborious, and expensive [15]. Recently next generation sequencing technology in combination with microarray methodology [16] has been demonstrated to be a fast and reliable tool for analysis of large genes such as *NEB*[15]. Patients are usually compound heterozygous for two different mutations [17]. The mechanisms leading to the alteration of muscle structure or rod formation are largely unknown.

Nemaline bodies are the pathologic hallmarks of congenital NM, even if these structures may sometimes be found associated with other conditions [18]. These are protein aggregates staining red with the modified Gomori trichrome technique. They can appear within the fibres as fine isolated/diffuse structures, compact subsarcolemmal clusters, or both [19]. On electron microscopy, nemaline bodies are electron dense and generally measure 1–7 μm in length and 0.3-2 μm in width. Due to their structural continuity with the Z-disk, and their resemblance to Z-disk lattice pattern, they are considered to be lateral expansions of the Z-disk [20-22]. In the case of nebulin mutation the rod formation could be due to a defect of the nebulin C-terminal, and serine-rich (SH3) domains. Concordantly, the nebulin C-terminal region, or part of it, may extend into the Z-disk [23]. Another common histologic finding of NM is type 1 predominance or type 1 uniformity [21,22]. Based on observations from consecutive muscle biopsies done in the same patient, a substitution of type 2 to type 1 fibres has been suggested to occur with increasing age [19,24]. All congenital NM patients seem to present a homogeneous morphological phenotype characterised by the presence of rods and type 1 predominance. However the largest series reporting on histologic NM findings were published before the identification of specific NM genes or they included patients harbouring mutations in other genes [22,25]. For this reason it is difficult to assess the existence of specific genotype-morphological phenotype correlations in the nine genetically identified forms of NM. A systematic morphological analysis of each entity is therefore pivotal in order to reveal pathogenetic mechanisms.

With the aim of characterising different patterns of muscle involvement, defining the relationship between morphological changes, genotype, and disease severity, we describe muscle morphology and functional studies of a large cohort of clinically heterogeneous *NEB*-mutated NM patients.

## Material and methods

### Patients

Fourteen patients from 13 unrelated families from France, the French Antilles, and Argentina were included in the present study. Patients were classified into three groups according to their clinical disease severity. P4 and P5 are brothers. P1 to P5 (Group 1) presented a severe/lethal congenital myopathy leading to death in the first days of life. Their muscle biopsy was performed between 2 days and 15 days of life. P6 to P9 presented an intermediate congenital myopathy and a biopsy effectuated between 2 and 10 months (Group 2); P10 to P14 presented typical or mild (P13, and P14) nemaline myopathy and a muscle biopsy performed during childhood or adolescence/early adult life (6 months-21 years; Group 3). The clinical data of these patients were systematically retrieved and retrospectively analysed. Patients were personally examined by one of 6 of the authors. Clinical and genetic characterization of P1, P2, P4, and P5 has been previously reported [4,15,26].

### Mutation analysis

Patients or parents gave informed consent for the genetic analysis according to French legislation (Comité de Protection des Personnes Est IV DC-2012-1693). Genomic DNA was extracted from blood by standard methods. As nemaline myopathy (NM) is genetically heterogeneous and as the immense size of the nebulin gene significantly impedes classical sequences approaches [12], we performed exome sequencing on 5 μg of genomic DNA from the patients and their parents as in Böhm et al. [15].

Exome sequencing was performed at the BGI (Shenzhen) on a Hiseq 2000 (Illumina) by using the Agilent 44 M v2 SureSelect Exon enrichment kit. Variant calling was done with the SOAP software. Variants filtering and prioritization were performed by comparison with SNP databases and with the VaRank program [27]. We discarded polymorphisms with a minor allele frequency (MAF) of more than 0.5% and excluded all variants with a frequency <20% of the total reads for a specific position. Additionally dHPLC and Sanger sequencing was performed in 7 patients as reported in Lehtokari et al. [17]. The mutations are reported according to the coding sequence of the nebulin cDNA reference sequence NM_001164508.1, and its translation.

#### RT-PCR

RNA was extracted from muscle biopsies with TRI-Reagent (Sigma), and cDNA was reverse transcribed using the SuperScript II Reverse Transcriptase (Invitrogen) and random hexamer primers. The PCR fragments of selected cDNA regions were cloned into the pGEM-T easy vector (Promega) and transformed into E. coli DH5alpha cells. Plasmid DNA was then extracted from single colonies and Sanger sequenced.

### Morphological studies

An open muscle biopsy was performed in all patients after informed consent. Age at biopsy varied from 29 weeks of adjusted gestational age to 21 years. The biopsied muscle is reported in Table 1 and was deltoid in 9 patients (P1, P2, P3, P5, P6, P9, P10, P13, and P14) and vastus lateralis in 5 (P4, P7, P8, P11, and P12). In order to make a precise and comparative study of muscle biopsy findings in Group 1 we standardized the age of new-borns calculating their ‘gestational adjusted age’ as described in Shichiji et al. [28] (Table 1). Samples were analysed in our research laboratory at the Myology Institute in Paris or in the Neuropathology laboratory of FLENI Institute and Garrahan Hospital in Buenos Aires, Argentina. For conventional histochemical techniques 10 μm thick cryostat sections were stained with haematoxylin and eosin (H&E), modified Gomori trichrome (mGT), Periodic acid Schiff technique (PAS), Oil red O, reduced nicotinamide adenine dinucleotide dehydrogenase-tetrazolium reductase (NADH-TR), succinic dehydrogenase (SDH), cytochrome c oxidase (COX), and adenosine triphosphatase (ATPase) preincubated at pH 9.4, 4.63, 4.35. Digital photographs of each biopsy were obtained with a Zeiss AxioCam HRc linked to a Zeiss Axioplan Bright Field Microscope and processed with the Axio Vision 4.4 software (Zeiss, Germany). The fibre type pattern was determined by counting 1000 fibres from each patient in ATPase 9.4 and 4.35 reactions, and by calculating the percentage of type 1 and type 2 fibres.

**Table 1:** Clinical, laboratory, and genetic features of patients

**Patient sex current age**	**Ethnic origin consanguinity**	**Age at onset (Gestational age)**	**Biopsied muscle Age at biopsy**	**Morphological methods (Functional studies)**	**Clinical phenotype**	**Permanent mechanical ventilation/age**	**NEB mutation: nucleotide/protein change**	**Effect of the mutations**	**Reference**
P1, F, deceased 10 days	French Caucasian, Yes	Antenatal (38 weeks)	Deltoid 2 days	IHC, IF, EM (Yes)	Group 1/Severe congenital nemaline myopathy Polyhydramnios, fœtal akinesia. Severe global hypotonia, respiratory distress, arthrogryposis, hip hyperlaxity, club feet and dysmorphic features.	Yes From birth	ex45; c.5574C > G; p.Tyr1858Stop; int122; c.19101 + 5G > A; p.Leu6333_Glu6367del	exon 45: nonsense mRNA decay (by RTPCR) exon 122: skipping confirmed by RTPCR and sequencing cDNA	Böhm et al., [15]
P2, F, deceased at 1 month	French Jewish (Ashkenazi) Yes	Antenatal, (36 weeks)	Deltoid 5 days	IHC, EM (No)	Group 1/Severe congenital nemaline myopathy Polyhydramnios, fœtal akinesia. Severe global hypotonia, respiratory distress, arthrogryposis, club feet and dysmorphic features.	Yes From birth	homozygous deletion of exon 55	Ashkenazi founder mutation; deletion of exon 55	Lehtokari et al., [26]
							c.7432 + 1916_7535 + 372del p.Arg2478_Asp2512del		
P3, M, deceased at 6 days	French Caucasian, No	Antenatal (38 weeks)	Deltoid 6 days	IHC, EM (No)	Group 1/Severe congenital nemaline myopathy Polyhydramnios, fœtal akinesia. Severe global hypotonia, respiratory distress, arthrogryposis, club feet, and dysmorphic features.	Yes From birth	ex86 (triplicated region); c.13066delT; p.Tyr4356Thrfs*8 ex110; c.17535G > A; p.Glu5845Glu	exon 86: frameshift mutation leading to either truncation or degradation. exon 110: splice site mutation	Present paper
P4, M, deceased at 5 days, Brother of P5	French Caucasian, Yes	Antenatal (29 weeks)	Vastus lateralis 3 days	IHC (No)	Group 1/Severe congenital nemaline myopathy Polyhydramnios, fœtal akinesia. Severe global hypotonia, absence of spontaneous movements at birth, respiratory distress, macrosomy and macrocephaly.	Yes From birth	ex177; c.24686_24687del; p.Glu8229Glufs*18	both mutations truncating/degrading	Pelin et al., [4] Lehtokari et al., [17]
							ex163; c.23420_23421del; p.Arg7807Serfs*16		
P5, M, deceased at 29 days, Brother of P4	French Caucasian, Yes	Antenatal (36 weeks)	Deltoid 15 days	IHC, EM (No)	Group 1/Severe congenital nemaline myopathy Polyhydramnios, macrosomy Severe global hypotonia, respiratory distress, reduced spontaneous movements, ptosis, arthrogryposis, hypetrichosis, and macrocephaly.	Yes From birth	ex177; c.24686_24687del; p.Glu8229Glufs*18	both mutations truncating/degrading	Pelin et al., [4] Lehtokari et al., [17]
							ex163; c.23420_23421del; p.Arg7807Serfs*16		
P6, M, deceased at 5 months	French Caucasian, No	Birth (39 weeks)	Deltoid 9 weeks	IHC, IF, EM (No)	Group 2/Intermediate congenital nemaline myopathy Hypotonia and poor spontaneous movements at birth. At one month respiratory distress and deglutition problems. Elongated face. High-arched palate. Low-set ears. Facial diplegia.	Yes From 1 month	ex6; c.300dup; p.Tyr101fs*5 int49, c.6496-G > A, p.2166_2234del	exon 6: truncating/degrading exon 50 skiping by RT and cDNA sequencing	Present paper
P7, F, deceased at 2 and half yrs	African, Yes	11 days (39 weeks)	Vastus lateralis 6 months	IHC, IF, EM (Yes)	Group 2/Intermediate congenital nemaline myopathy. Apparently normal at birth. Successively hypotonia and deglutition problems. At 1.5 months development of progressive respiratory failure followed by recuperated cardiac arrest.	Yes From 2 months	homozygous 177; c.24735-24736DelA_fsx1 is this c.24735_24736del (AG) p.Arg8245fs*1	truncating/degrading	Present paper
P8, M 5 yrs	Argentinian, No	6 months (40 weeks)	Vastus lateralis 6 months	IHC, EM (Yes)	Group 2/Intermediate congenital nemaline myopathy Hypotonia, motor delay. High-arched palate. Proximal and distal muscle weakness. Retractions of fingers. Mild hyperlaxity. At 1 year development of progressive respiratory involvement necessitating tracheostomy.	Yes From 1 month	ex139; c.20928G > T; p.Gly6976Gly	RTPCR and Sanger sequencing of cDNA showed that instead of 105 nt, exon 139 contains only 34 nt: frameshift and a premature stop codon. Exon 172; truncating/degrading	Present paper
							ex172; c.24269del p.Arg8090fs*54		
P9, M, 11 yrs	French Caucasian, Yes	Birth (39 weeks)	Deltoid 10 months	IHC, IF, EM (Yes)	Group 2/Intermediate congenital nemaline myopathy Shortly after birth severe respiratory failure. Tracheostomy and gastrostomy at five months. Facial diplegia, drooling, deglutition problems. Axial hypotonia and weakness of all limb muscles.	Yes From 5 months	homozygous ex174; c.24440_24441insGTCA, p.Pro8148Serfs*15	truncating/degrading	Present paper
P10, M, 17 yrs	French Caucasian, No	6 years (At term)	Deltoid 6 years	IHC, IF, EM (Yes)	Group 3/Typical congenital nemaline myopathy. Global hypotonia, deglutition problems. Proximal muscle weakness. Facial diplegia. Nasal voice. Mild respiratory insufficiency treated with discontinuous non-invasive ventilation.	No	int43; c.5343 + 5G > A p.Arg1747_Thr1778del ex153; c.22273del p.Val7425Serfs49*	intron 43: a splice site mutation exon153: truncating/degrading	Present paper
P11, M, 19 years	French Antillean No	1 yrs (At term)	Vastus lateralis 6 yrs	IHC, IF, EM (Yes)	Group 3/Typical congenital nemaline myopathy. Hypotonia and feeding difficulties. Delayed motor milestones. Facial weakness with open mouth. Axial and limb girdle proximal weakness. Mild respiratory involvement treated with discontinuous non-invasive ventilation.	No	ex175; c.24579G > A, p.Ser8193Ser ex119; c.18676C > T, p.Gln6226*	ex175: a splice site mutation ex119: a nonsense mutation (truncating/degrading)	Present paper
P12, M, 20 yrs	French Caucasian, No	2-3 years (At term)	Vastus lateralis 6 years	IHC, EM (No)	Group 3/Typical congenital nemaline myopathy. Difficulties in running and rising stairs. Facial weakness with open mouth. Mild upper and lower limb girdle weakness.	No	int155; c.22591-3C > G; p.7531Val_Ser7564del; ex148; c.21796_21810delinsT; p.Pro7266fs*30	intron 155: a splice site mutation exon 148: truncating/degrading	Present paper
P13, F, 52 yrs	French Caucasian, No	6 yrs (At term)	Deltoid 18 years	IHC, EM (No)	Group 3/Mild congenital nemaline myopathy. Difficulties in sport activities in school. Bilateral pes cavus. Presence of mild upper girdle musle weakness. Diffuse muscle pain.	No	ex69; c.10043_10046del, p.Val3348Alafs *43 ex49; c.6388G > C p.Ala2130Pro	ex69: truncating/degrading ex49: missense on the acting binding site	Present paper
P14, F, 37 yrs	French Caucasian, No	2 yrs (At term)	Deltoid 21 yrs	IHC, IF, EM (Yes)	Group 3/Mild congenital nemaline myopathy. Frequent falls. Difficulties in running , rising stairs. Jaw contractures. Nasal voice. Elongated face. Respiratory involvement. Axial weakness with difficulties in neck flexion. Asymmentrical distal weaakness with foot drop (right > left). Proximo-distal weakness.	No	int17; c.1569 + 1G > A p.His491_Asp523del	intron 17: a splice site mutation	Present paper
								exon 176: truncating/degrading	
							ex176; c.24606del p.Ala8203Glnfs*13		

Based on our experience, and the fibre type proportion reported in the literature regarding the muscle analysed, we considered type 1 fibres predominance to be present when there were more than of 60% type 1 fibre in deltoid muscles, and more than 40% in vastus lateralis muscle [29]. Fibre type distribution in G1 patients was analysed comparing the data on muscle fibre patterns during the main phases of skeletal muscle development obtained from individual with no neumuscular disorder [30,31]. Moreover, where possible, we analysed fibre type proportion in age-matched control biopsies corresponding to G2, and G3 patients.

For the analysis of the proportion of fibres with rods, 800–1000 fibres of the muscle sections of each patient were analysed, and the percentage of fibres appearing with and without rods on the total number of fibres of a muscle sections was calculated; four consecutive, non-overlapping fields were counted. In addition, a classification of the rods and their pattern was effectuated. We defined the rods as being cytoplasmic when localised mainly inside the fibres sparing the subsarcolemmal areas, scattered when they were randomly distributed in the muscle fibre, diffuse, when several small rods were distributed across the whole fibres homogenously occupying the majority of their area, central when distributed mainly in the centre of the cytoplasm, and subsarcolemmal when they were localized in a compact manner close to the fibre membrane (as clusters). We also evaluated the shape of nemaline bodies being mainly globular/ovoid, squared, or elongated.

### Immunohistochemistry and immunofluorescence

Frozen muscle samples for immunohistochemical and immunofluorescence analyses were available for 7 patients (P1, P6, P7, P9, P10, P11, and P14). Myosin heavy chain fast (NCL-MHCf, Novocastra Laboratories, Newcastle Upon Tyne, United Kingdom), myosin heavy chain slow (NCL-MHCs, Novocastra Laboratories, Newcastle Upon Tyne, United Kingdom), myosin heavy chain developmental (NCL-MHCd, Novocastra Laboratories, Newcastle Upon Tyne, United Kingdom), myosin heavy chain neonatal (NCL-MHCn, Novocastra Laboratories, Newcastle Upon Tyne, United Kingdom). Antibodies were visualized using immunoperoxidase techniques [28]. Myosin alpha and beta-slow heavy chain, fast 2A heavy chain, and 2X myosin heavy chain (BA-D5, SC-71, and 6H1, Developmental Studies Hybridoma Bank, University of Iowa, Iowa City, USA) immunofluorescence were assessed on 10-μm-thick cryosections over night at 4°C. Subsequently, sections were incubated with appropriate conjugated secondary antibodies for one hour (Alexa Fluor-488 green goat anti-rabbit antibody, and Alexa Fluor-594 red goat anti-mouse antibody, Molecular Probes, Cergy Pontoise, France). A set of control slides was prepared with omission of the primary antibodies.

### Electron microscopy

Detailed electron microscopy analysis was prospectively performed in thirteen patients. Small muscle specimens were fixed with glutaraldehyde (2.5%, pH 7.4), post fixed with osmium tetroxide (2%), dehydrated and embedded in resin (EMBed-812, Electron Microscopy Sciences, USA). Ultra-thin sections from at least three small blocks from each patient were stained with uranyl acetate and lead citrate. The grids were observed using a Philips CM120 electron microscope (80 kV; Philips Electronics NV, Eindhoven, The Netherlands) and were photo documented using a Morada camera (Soft Imaging System, France).

### Muscle contractility experiments

To investigate whether the contractile performance is affected in muscle biopsies from patients with mutations in the nebulin gene, we performed skinned muscle fibre contractility experiments. Small strips were dissected from muscle biopsies of patients P1 (Group 1), P7, P8 and P9 (Group 2), and P10, P11 and P14 (Group 3) and were skinned overnight as described previously [32]. The skinning procedure renders the membranous structures in the muscle fibres permeable, which enables activation of the myofilaments with exogenous Ca^2+^. Preparations were washed thoroughly with relaxing solution and stored in 50% glycerol/relaxing solution at -20°C. Small muscle preparations (cross-sectional area ~0.002 mm^2^) were dissected from the skinned strips, and were mounted using aluminum T-clips between a length motor (ASI 403A, Aurora Scientific Inc., Ontario, Canada) and a force transducer element (ASI 315C-I, Aurora Scientific Inc., Ontario, Canada) in a single fibre apparatus (ASI 802D, Aurora Scientific Inc., Ontario, Canada) that was mounted on an inverted microscope (Zeiss Axio Observer A1). Sarcomere length was set using a high speed VSL camera and ASI 900B software (Aurora Scientific Inc., Ontario, Canada). Mechanical experiments were performed at a sarcomere length of ~2.2 μm, a length selected to minimize force differences due to shorter thin filaments in fibres from nemaline myopathy patients with nebulin mutations [32]. Fibre width and diameter were measured at three points along the fibre and the cross-sectional area was determined assuming an elliptical cross-section. Two different types of bathing solutions were used during the experimental protocols: a relaxing solution (40 mM BES; 10 mM EGTA; 6.86 mM MgCl_2_; 5.96 mM Na-ATP; 3.28 mM K-propionate; 33 mM creatine phosphate; 1 mM DTT; 0.5 mM PMSF; 0.2 mM Leupeptin; 0.05 mM E64) and an activating solution (40 mM BES; 10 mM CaCO_3_-EGTA; 6.64 mM MgCl_2_; 6.23 mM Na-ATP; 2.1 mM K-propionate; 15 mM creatine phosphate; 1 mM DTT; 0.5 mM PMSF; 0.2 mM Leupeptin; 0.05 mM E64). The temperature of the bathing solutions was controlled by a TEC controller (ASI 825A, Aurora Scientific Inc. Ontario, Canada). Muscle preparations were mounted in a relaxation solution at 1°C. Subsequently, the muscle preparations were pre-activated by switching to an activation solution at 1°C. In that way the myofibres are loaded with calcium, but no force is generated. By rapid switching to an activation solution at 20°C, the fibres were activated and force was generated. When the force trace reached a plateau, the muscle fibres were slacked to 70% of their original length followed by a rapid restretch to the original length after 30 milliseconds. This procedure allows the force to redevelop from zero [33]. The rate of tension redevelopment was calculated by fitting a bi-exponentional through the force redevelopment curve. The first-order rate constant *k*_*1*_ reflects crossbridge cycling kinetics and was therefore used in the analyses [34].

### Myosin heavy chain composition of bundles used for contractility experiments

For determination of the myosin heavy chain isoform composition of the muscle fibre preparations we used specialized SDS-PAGE [32]. In brief, muscles fibres were denatured by boiling for 2 minutes in SDS sample buffer. The stacking gel contained a 4% acrylamide concentration (pH 6.7), and the separating gel contained 7% acrylamide (pH 8.7) with 30% glycerol (v/v). The gels were run for 24 h at 15°C and a constant voltage of 275 V. Lastly, the gels were silver-stained, scanned, and analysed with One-D scan EX software (Scanalytics Inc., Rockville, MD, USA).

The ethical committee of La Pitié-Salpêtrière Hospital (CCPPRB) approved this study.

## Results

### Clinical findings

Five patients were female and 9 were male. Clinical summary, laboratory features, a complete list of morphological methods, functional studies applied to muscle biopsies, and genetic characterization of all patients is provided in Table 1. Patient 6 and 7 were classified to be a part of intermediate congenital nemaline myopathy group because they were breathing and moving at birth. However, these patients developed soon after birth a severe clinical picture and they were never able to achieve respiratory independence and/or ambulation. They eventually deceased at 5 months, and 32 months respectively. They therefore present a phenotype in between a severe and an intermediate congenital nemaline myopathy.

### Molecular data

To identify the genetic cause in this cohort of patients, we performed exome enrichment and sequencing on genomic DNA from the patients and their parents. Exome sequencing allows a rapid and parallel screening of most human genes, and is suitable and efficient for the diagnosis of neuromuscular diseases and the analysis of large genes such as *NEB*, frequently mutated in NM [15]. This approach also covers any newly discovered gene for the disorder. For all patients presented here we found known or novel variations in the *NEB* gene. These changes were confirmed by Sanger sequencing, and their familial segregation validated when parent DNA available. In all the patients, at least one of the two compound heterozygous mutations was a truncating mutation (frameshift or nonsense mutation) leading to protein truncation or degradation, or both (Table 1). The second mutations were, either a frameshift mutation, deletion of several amino acids in frame (splice site mutations), a non-conservative missense change close to an actin binding site (P13), or synonymous variants in P3, P8, and P11 that all impacted on splicing. Most of the mutations (18) affected ubiquitously expressed exons, while six were in the alternatively spliced exons and one in an exon of the triplicated region of eight exons. Overall, most mutations were predicted to lead to a truncated or absent protein. There was no obvious correlation between the type of mutation or its location on the protein and the clinical severity.

## Morphological findings

### Histological and histochemical features

#### Group 1

**Figure 1:**
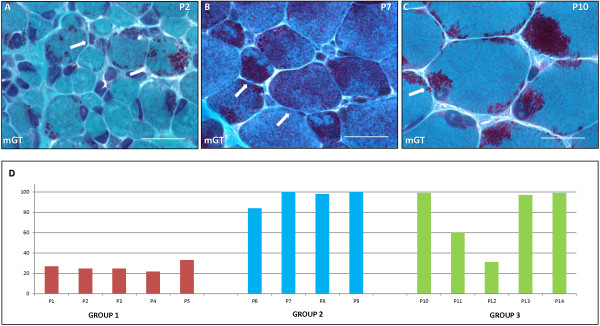
**Light microscopy and percentage of fibers with rods.** Modified Gomori trichrome stainings. **A** and **B**. Nemaline bodies in muscle biopsies from P2 (G1) and P7 (G2) have a rounded/ovoid shape. They are present in both normal size, and atrophic fibres (indicated by arrows). **C**. Nemaline bodies in muscle biopsy from P11 have an elongated shape (indicated by arrows) and they are localized in subsarcolemmal and perinuclear areas. Scale bars represent 20 μm for mGT stainings. **D**. Representation of percentage of rod occupied fibres in Group 1 (red), Group 2 (blue), and Group 3 (green). See text for explanations.

A similar morphological pattern characterised by marked fibre size variability was noted in all biopsies from G1. We constantly identified two populations of fibres; the first characterized by muscle fibres of predictably normal or slightly augmented size, and the second one consisting of severely atrophic fibres (Figure 1A; arrows). Small rounded/globular inclusions staining red by the mGT, corresponding to nemaline bodies, were present in less than half of muscle fibres (Figure 1A). Nemaline bodies occupied both normal-sized and atrophic fibres (Figure 1A). They presented a dispersed or, more often, a subsarcolemmal distribution (Figure 1A). Some atrophic fibres appeared completely occupied by nemaline bodies. The percentage of muscle fibres harbouring rods was to 22% to 33% (Figure 1D, Group 1, red). The oxidative enzyme reactions revealed more than 30% of fibres presenting uneven staining of the intermyofibrillar network (not shown). The latter did not always correspond to the areas occupied by nemaline bodies, suggesting the presence of some degree of sarcomeric disruption. ATPase techniques did not reveal type 1 fibre predominance in any of the patients in G1. The majority of fibres staining differently from type 1 are probably undifferentiated fibres (not shown).

#### Group 2

The morphological pattern found in this group was heterogeneous compared with G1 biopsies. While in P6, and P7 samples we noticed the presence of two populations of muscle fibres (predictably normal size and severely atrophic) (Figure 1B; arrows), P8, and P9 showed a mild variation of fibres size without any particular topography (not shown). Conversely to G1 nemaline bodies were present in the vast majority of fibres (mean: 95% to 100%; Figure 1D, Group 2, blue) and presented a variable shape varying from oval to elongated. The oxidative enzyme reactions revealed some alteration of the intermyofibrillar network, probably corresponding to rod accumulation or myofibrillar disorganization with oxidative techniques. ATPase techniques showed type 1 fibre predominance in P6, and P8. Type 1 uniformity was noted in P7 and P9.

#### Group 3

In this group we noticed mild variation of fibre size except in P12 where some atrophic rounded fibres were identified (not shown). Nemaline bodies presented a constant elongated shape and formed well separated clusters both in subsarcolemmal and cytoplasmic areas (Figure 1C). There was a large variability in the percentage of fibres harbouring rods. While P10, P13 and P14 presented rods in almost all fibres (97-99%), P11 had 60% and P12 31% of fibres with rods, respectively (Figure 1D, Group 3, green). ATPase techniques showed almost complete type 1 uniformity in G3 muscle biopsies. The areas of muscle fibres containing rods lacked ATPase staining.

### Summary

Overall, severe NM was associated with a fibre size variability, presence of rods in about 1/3 of fibres, and a high percentage of undifferentiated fibres. By contrast a higher percentage of fibres with rods, and a type 1 fibre predominance/uniformity was noted in the intermediate and typical NM patients. The amount of nemaline bodies seems to be inversely related to clinical severity.

### Immunohistochemistry and immunofluorescence

#### Group 1

In P1 we identified many fibres expressing developmental, neonatal, fast and/or slow myosin. Immunofluorescence studies showed absence of type 2X myosin.

#### Group 2

In P6 we identified many fibres co-expressing slow and fast myosins. Developmental and neonatal myosins were expressed in a minority of fibres. In P8 we identified occasional fibres expressing developmental myosin and less than 5% of fibres expressing neonatal myosin; there was partial co-expression of fast and slow myosins. P9 showed unique expression of slow myosin both in immunohistochemical, and immunofluorescence studies.

#### Group 3

P10, P11, and P14 showed slow myosin uniformity using both techniques. No fibres expressing developmental and/or neonatal myosins were noted.

### Electron microscopy

#### Group 1

**Figure 2:**
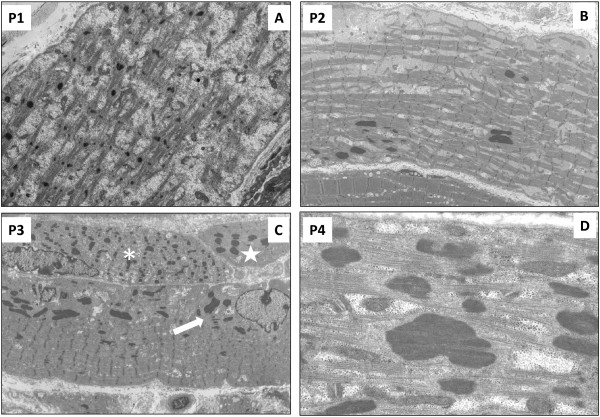
**Electron microscopy for group G1-severe NM. A**. P1. Sarcomeric structure is completely disrupted. Fragment of sarcomeres are intermingled with amorphous material containing organelles and glycogen granules. **B**. P2. Scattered globular ovoidal nemaline bodies are found inside a fiber showing partial sarcomeric alteration. **C**. P3. Presence of three muscle fibres presenting different degree of alterations. Nemaline bodies are found in subsarcolemmal areas (indicated by an arrow). The fibre above the first one shows sarcomeric disarray and thicknened Z-lines probably leading to globular rods formation (indicated by an asterisk). A small atrophic fibre is completely invaded by rounded nemaline bodies (indicated by a star). **D**. P4. Globular nemaline bodies present thin filaments coming out from the thinner edges. Original magnification: **A**. 11,000x. **B**. 7,000x **C**. 8,400x **D**. 51,000x.

The prominent ultrastructural finding in all G1 patients was the diffuse myofibrillar dissociation. The myofibrils appeared thinner, and smaller than in age-matched controls (Figure 2A). The latter suggested either a defect in sarcomeric structure establishment either the lost of it. Remnants of sarcomeres were intermingled with organelles (e.g. mitochondria) or glycogen granules (Figure 2A). Globular/ovoid nemaline bodies were scattered or distributed in subsarcolemmal and perinuclear areas (Figure 2B, and C). Some fibres were completely occupied by them (Figure 2C). At higher magnification globular/ovoid rods presented thin filaments projecting from their thinnest edges (Figure 2D).

#### Group 2

**Figure 3:**
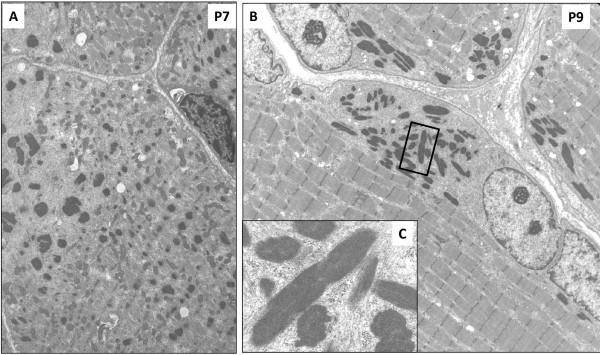
**Electron microscopy for group G2-intermediate NM. A**. P7. Globular ovoid nemaline bodies associated with partial sarcomeric disarray strongly resembling the pathologic picture of G1. **B**. P9. Presence of elongated nemaline bodies in perinuclear and subsarcolemmal areas. The latter are well separated from well-preserved sarcomeric structure. **C**. P9. Higher magnification of an elongated nemaline body. The typical periodic net structure composing rods is clearly recognisable. Thin filament spread out of the thinnest edges of rods. Sparse thin filaments are found around the rods intermingled with glycogen granules. Original magnification: **A**. 11,000x **B**. 6,400x **C**. 94,000x.

This group presented a milder degree of myofibrillar dissociation accompanying dispersed or clustered nemaline bodies (Figure 3A, and 3B). Rods showed the typical lattice structure resembling Z-disc material at very high magnification (Figure 3C). In P9 we noticed the presence of some typical cytoplasmic bodies with a dense core, and a clear halo of fine filaments (not shown).

#### Group 3

**Figure 4:**
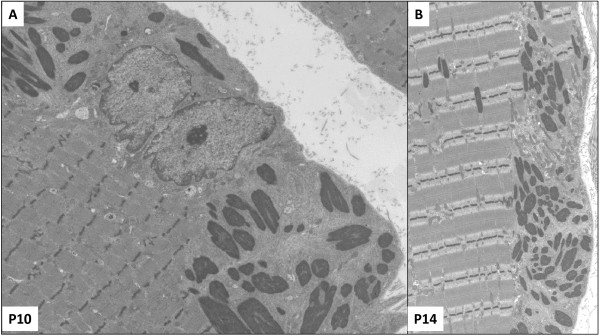
**Electron microscopy for group G3-typical-mild NM. A**. P10, G3. Presence of globally preserved sarcomeric structure associated with a cluster of elongated nemaline bodies in the subsarcolemmal areas of muscle fibres. P10. **B**. P14. Well-delimited clusters of rods surrounded by thin filaments. Original magnification: **A**. 9,000x **B**. 8,200x.

We found a homogenous picture characterised by the presence of well-separated clusters of subsarcolemmal (Figure 4A), perinuclear, and less often cytoplasmic nemaline bodies (Figure 4B). The rods were always surrounded by thin filaments and amorphous material. The sarcomeric structure was overall conserved (Figure 4A, and 4B).

### Summary

Taken together, our results suggest that myofibrillar dissociation correlated with clinical severity.

### Muscle contractility experiments

**Figure 5:**
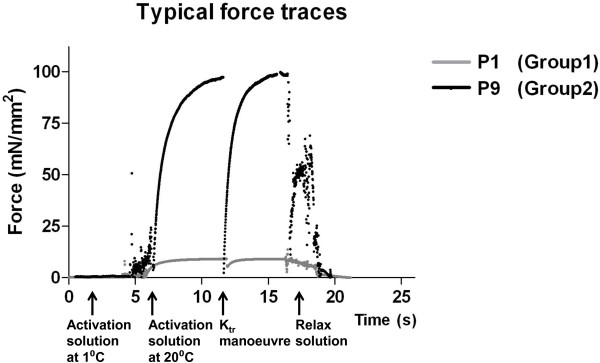
**Typical force traces of skinned myofibers from nemaline myopathy patients with nebulin mutations.** Typical examples from force traces of a skinned muscle preparation from P1 from Group1 (in grey) and P9 from Group2 (in black). Myofibres are pre-activated by exposure to an activation solution at 1°C. By rapid switching to an activation solution at 20°C, the fibers are activated and force is generated. When the force trace reaches a plateau, the myofibres are slacked to 70% of their original length followed by a rapid restretch to the original length after 30 milliseconds (K_tr_ manouvre). When force generation has reached a plateau again, the muscle preparation is exposed to a calcium-free solution to induce relaxation.

The maximal force generation capacity of the muscle fibre preparations was normalized to their cross-sectional area (i.e. maximal active tension) (Figure 5). An overview of the maximal active tension and the rate of tension redevelopment of the muscle fibres are summarized in Table 2. Data are presented as mean ± SEM.

**Table 2:** **Muscle contractility data of permeabilized fibers from nemaline myopathy patients with *NEB *** **mutations**

	**P1**	**P7**	**P8**	**P9**	**P10**	**P11**	**P14**
**Maximal active tension (mN/mm**^**2**^**)**	8.1 ± 1.5	18.8 ± 2.7	76.9 ± 10.0	90.9 ± 10.5	55.5 ± 7.1	102.2 ± 21.2	61.4 ± 12.2
**Rate of tension redevelopment *(k***_***1 ***_**(s**^**-1**^**))**	5.1 ± 0.4	4.5 ± 0.7	5.0 ± 0.5	3.4 ± 0.2	3.6 ± 0.2	4.0 ± 0.6	3.8 ± 0.5

### Myosin heavy chain analyses

The myosin heavy chain (MHC) gel electrophoresis experiments revealed that muscle preparations from biopsies from patient P1 contained both neonatal isoforms as well as type 1 and type 2A isoforms: (9.2 ± 2.8% MHC neonatal; 40.0 ± 3.2% MHC type 1; 50.8 ± 1.3% MHC type 2A). Muscle preparations from biopsies of P7 and P8 (group 2) contained both MHC type 1 and MHC type 2A isoforms: P7 (66.6 ± 6.6% MHC type 1 and 33.4 ± 6.6% MHC type 2A) and P8 (40.8 ± 12.0% MHC type 1 and 59.2 ± 12.0% MHC type 2A), respectively. All other patient biopsies (P9, P10, P11 and P14) showed exclusively myosin heavy chain type 1 isoforms.

## Discussion

In our tertiary Center for Neuromuscular Disorders we perform a detailed clinical, morphological, and genetic analysis of large cohorts of patients presenting NM. Due to the genetic heterogeneity of NM, and the difficulties encountered in the molecular screening of the ‘giant’ *NEB* gene [15,35], in France and Finland an integrated approach combining next generation sequencing and dHPLC/Sanger sequencing was set-up. Our strategy allowed the identification of ten new families harbouring *NEB* mutations. All patients presented autosomal recessive pattern of inheritance and either homozygous or compound heterozygous pathogenic variants. Our results confirm that *NEB* is one of the most frequently mutated NM genes, accounting for almost half of the genetically identified NM patients screened for the known genes associated with NM [3-11].

We undertook a detailed clinical histological, and, when possible, muscle functional analysis in a cohort of fourteen *NEB*-mutated subjects whose muscle biopsy was available in our laboratory. We comment on relevant findings encountered.

*NEB*-mutated patients revealed a wide pathological spectrum and showed recurrent morphological pattern with some overlap among the clinical groups. Lethal/severe NM subjects (G1) presented: high degree of myofibrillar dissociation and smallness revealed by electron microscopy, scattered globular/ovoid nemaline bodies occupying one third of muscle fibres, and absence of type 1 predominance with myosins ATPases techniques (Figure 2). Intermediate congenital myopathy patients (G2) showed features similar to G1 in P6 and P7, even though the presented higher percentage of rods and type 1 predominance, and well-separated clusters of rods associated with type 1 predominance or uniformity in the other patients. It is noteworthy that P6 and P7 deceased at 5 months, and 2 and a half year, respectively. For this reason these patients could be considered as a ‘*Longer survivor severe congenital NM*’ subgroup due to their ‘midway’ clinical and morphological features between G1 and G2. Group 3 had a preserved sarcomeric structure with clusters of elongated rods invariantly associated with type 1 predominance/uniformity.

In summary we show a large pathological spectrum ranging from severely damaged sarcomeres with scattered nemaline bodies to globally preserved muscle with clusters of well separated rods occupying the majority of myofibres. Interestingly the degree of sarcomeric disruption directly related to clinical severity whereas the number of rod invaded fibres seemed to be inversely correlated.

We undertook this study to search for genotype-phenotype correlations. Taken together, the vast majority of *NEB* mutations are predicted to lead to a degradation and or denaturation of many nebulin isoforms. Markedly reduced amounts of nebulin in muscle samples from patients homozygous for exon 55 deletions have been reported previously [36]. P2 in this study is homozygous for the same exon 55 deletion, resulting in an in-frame deletion of 35 amino acids, and subsequent protein degradation. Nonsense mRNA-mediated decay was demonstrated by RT-PCR in P1, who carries a heterozygous nonsense mutation in exon 45. P1 is also heterozygous for a splice site mutation in intron 122, which was shown to cause exon 122 skipping, resulting in in-frame deletion of 35 amino acids [15]. Splice site mutations are, however, often “leaky”, i.e. some transcripts are spliced correctly, whereas others are incorrectly spliced. Therefore, it seems plausible that P1, as well as the other patients with splice site mutations (P3, P6, P8, P10, P11, P12 and P14) express small amounts of normal nebulin in their muscles. Markedly reduced amounts of nebulin in muscle have been reported in one patient compound heterozygous for a splice site mutation and a frameshift mutation in constitutively expressed exons [37]. The mutations in alternatively spliced exons (exons 174, 175, 176 and 177) only affect nebulin isoforms expressing these exons, leaving other isoforms unaffected. Consequently reduced amounts of nebulin and absence of some isoforms, precluding an appropriate thin filaments assembly, might be responsible for the drastic myofibrillar dissociation revealed in G1 [Figure 4]. Functional studies confirmed that G1 patients’ muscle fibers generate very low force. Although one could argue that age confounded our findings, previous work from our group revealed no major differences in the contractile performance of myofibres isolated from young (age 2–5 years) versus adult (age 20–30) human control biopsies [37]. Note that G1 patients’ muscle fibres expressed only very low levels of neonatal myosin heavy chain isoforms; such low levels are unlikely to account for the major loss of force in this patient. We therefore suggest that nebulin degradation/absence translates into sarcomeric disarray and/or altered actomyosin interaction. This could be responsible for low force generation producing global hypotonia, muscle akinesia, and arthrogryposis. Concordantly, a recent study reported that low levels of nebulin in skeletal muscle are probably responsible for the foetal akinesia and arthrogryposis sequence phenotype [36].

How nebulin deficiency results in nemaline bodies formation is yet to be understood. Some authors suggested that a truncated nebulin would disrupt myofibrillar connectivity leading to Z-disc displacement and, eventually, rods formation [38]. Analysis of nemaline bodies features in our cohort revealed differences in shape across the three groups. These tended to be globular/ovoid in G1, elongated in the other groups. The specificity of this finding is uncertain. What is more striking is that in P8, and P9 from G2 and in all G3 patients, the majority of fibres harboured rod clusters confined to subsarcolemmal and/or perinuclear areas. The myofibrillar structure surrounding them was overall preserved. Some unknown process could try to circumscribe the protein aggregates and avoid a perturbation of muscle contraction. This could explain why muscle fibers in G2 and G3 patients showed a better contractile performance, probably translating into a milder clinical phenotype. It is tempting to speculate that mutations encountered in these groups affect only specific nebulin isoforms, which is certainly true for patients P7, P8, P9, P11, and P14 who all have at least one mutation in an alternatively spliced exon. In this scenario, residual normal isoforms could allow a proper thin filament assembly, while the altered ones might be responsible for protein aggregation/rod formation. If this turned to be true, the presence of rods might be considered unrelated to muscle contractility disturbances. Following this reasoning we could imagine that an additive effect of degraded nebulin isoforms would determine clinical severity.

Type 1 predominance or type uniformity has been reported as a very common feature associated with NM, and many other structural congenital myopathies [39]. It is speculated that this is due to a disturbance of fibre differentiation before phase three of muscle differentiation (35th weeks) [40]. A severe *NEB* mutated family composed by two brothers has been reported as not having type 1 fibre predominance. However, biopsied muscle and age at muscle biopsy were not specified [41]. In the present study we performed type fibre distribution analysis on ATPase techniques. Surprisingly, all G1 patients failed to show type 1 predominance and constantly showed absence of 2B fibers. Immunostainings for different myosins isoforms (foetal neonatal, fast and slow) revealed a certain degree of myosin isoforms co-expression in numerous fibres suggesting the presence of undifferentiated fibers. This contrasted with the other groups where type 1 predominance/uniformity was present. This finding suggests that G1 *NEB*-mutated patients were not able to switch towards type 1 predominance due to a possible alteration of muscle maturation. It is tempting to speculate that aberrations in fibre-typing, and absence of type 2B seen with ATPase techniques are due to changes in isoforms imbalance more than related to age at muscle biopsy. Additionally the presence of high percentages of undifferentiated fibres encountered in G1 might turn out to be an important prognostic factor. In fact muscle biopsy analysis of new-borns presenting arthrogryposis and a pathological picture characterised by sarcomeric dissociation, scattered nemaline bodies absence of type 1 predominance and type 2B fibres with ATPases techniques might orientate toward *NEB* mutations. This is something distinctive from other severe form of congenital myopathies commonly showing type 1 predominance as a prominent feature [42]. In particular we recently demonstrated that *MTM1*-mutated boys presenting an extremely severe clinical phenotype all had type 1 predominance in their biopsies, regardless of the biopsied muscle, and the gestational age [28].

In conclusion, this study adds on the clinical, morphological and functional characterization of the most recurrent form of NM. We assessed morphological and functional heterogeneity in *NEB*-mutated NM patients and identified a correlation between disease severity on the one hand, and ultrastructural myofibrillar abnormalities and contractility on the other. We suggest that myofibrillar dissociation and smallness is a primary defect causing the disease while nemaline bodies could be due to a collateral mechanism.

## Competing interests

The authors declare that they have no competing interests.
